# Antibiotic Consumption and Resistance during a 3-Year Period in Sicily, Southern Italy

**DOI:** 10.3390/ijerph16132253

**Published:** 2019-06-26

**Authors:** Martina Barchitta, Annalisa Quattrocchi, Andrea Maugeri, Maria Clara La Rosa, Claudia La Mastra, Laura Sessa, Pasquale Cananzi, Giuseppe Murolo, Alessandro Oteri, Guido Basile, Antonella Agodi

**Affiliations:** 1 Department of Medical and Surgical Sciences and Advanced Technologies “GF Ingrassia”, University of Catania, 95123 Catania, Italy; 2 Regional Health Authority of the Sicilian Region, 90145 Palermo, Italy; 3 Department of General Surgery and Medical-Surgical Specialties, University of Catania, 95123 Catania, Italy; 4 AOU Policlinico-Vittorio Emanuele, 95123 Catania, Italy

**Keywords:** public health, surveillance, indicators, resistance rates, defined daily dose

## Abstract

Antimicrobial resistance (AMR) is one of the biggest issues facing global public health. In 2017, Italy adopted its first National Action Plan on Antimicrobial Resistance 2017–2020, which works through the synergy between national, regional, and local levels. In the framework of a Regional Action Plan on healthcare-associated infections and AMR prevention, the Sicilian Health Authority of the Sicilian Region, Southern Italy, has implemented a surveillance system of antibiotic consumption in hospitals, in the community, and of resistance rates (RRs) in hospital settings. Data on antibiotic consumption and on antibiotic RRs have been collected from 2015 to 2017 from pharmacies and laboratories of participating hospitals and from community, respectively. Data on antibiotic consumption showed that the most consumed antibiotics in hospitals were fluoroquinolones in 2015, penicillin in 2016, and beta-lactams in 2017. From 2015 to 2017, data on *Klebsiella pneumoniae* showed significant increasing RRs to all antibiotic classes, except to carbapenems. RRs of third-generation cephalosporins and carbapenems *Escherichia coli* showed significant decreasing trends. RRs of the other microorganisms did not change significantly during the study period. The results from the present study show that in Sicily, the use of antibiotics and RRs for selected microorganisms are at a high level. Immediate strategies are needed to decrease the inappropriate usage of antibiotics and control the spread of AMR.

## 1. Introduction

Antimicrobial resistance (AMR) is one of the ten threats identified by the World Health Organization (WHO) in 2019, since it affects modern healthcare and the effective prevention and treatment of an ever-increasing range of infections [1]. Recent estimates of the burden of AMR are very impressive with a total of 671,689 cases of infections with selected antibiotic-resistant bacteria occurring in 2015 in Europe, out of which 63.5% were associated with healthcare. The attributable mortality was 6.44 deaths per 100,000 population and the overall disability-adjusted life years rate was 170 per 100,000 population. Notably, this burden was highest in Italy and Greece than in other European countries [2,3]. The increasing of multidrug-resistant (MDR) microorganisms and of community-acquired and healthcare-associated infections (HAIs) are strictly connected [4]. However, although MDR bacteria are usually linked to HAIs, many resistant microorganisms have also been associated with community-acquired infections [5]. Indeed, a high number of MDR microorganisms have been isolated from common infections (e.g., urinary tract infections, pneumonia, tuberculosis, and gonorrhea) [6]. MDR gram-negative bacteria have been increasing over the last few decades [7]. Specifically, MDR gram-negative pathogens, such as *Klebsiella pneumoniae*, *Acinetobacter baumannii*, and other *Enterobacteriaceae* that produce extended-spectrum beta-lactamases (ESBL), have been shown to be involved in severe HAIs and frequently involved in outbreaks, especially in intensive care units [8,9,10,11].

AMR comes from different mechanisms, such as the acquisition of genes carried by plasmid or other genetic elements [4]. In addition to acquired resistance, bacteria including *Pseudomonas aeruginosa*, *Enterococcus faecalis*, and *Enterococcus faecium* have shown intrinsic resistance to common antibiotics which results in very virulent microorganisms that cause infections to become hard to manage [12]. Gram-positive bacteria are also associated with community-acquired infections. In this contest, methicillin-resistant *Staphylococcus aureus* (MRSA), which was at first only involved in HAIs and successively became wide spread in the community, is the most representative microorganism [5]. 

The inappropriate use of antibiotics and the large use of broad spectrum antibiotics, both in health care facilities and in the community, is one of the biggest causes of the emergence and propagation of AMR [13]. Furthermore, the inappropriate use of antibiotics in agriculture and in veterinary sectors contributes to AMR [14]. The increase of AMR also has a negative impact on the global economy as it leads to growing costs in healthcare (longer hospital stay, lower quality of medical procedures, treatment failure, and mortality) [15,16]. Another issue to be considered is the lack of new antibiotics, which compromises the success of therapy in all medical fields from surgery to chemotherapy, from severe infections in patients admitted in intensive care units to common infections in the community [17]. Scientific evidence shows how a rational use of antibiotics and a comprehensive infection prevention and control strategy is necessary in avoiding the selection and transmission of MDR [18], including hand hygiene and other infection control measures [19,20,21,22,23]. AMR represents a serious public health threat which needs immediate strategies to monitor and counteract the inappropriate use of antimicrobials. Great attention should be paid to fight AMR at the international, national, and local level. Therefore, multiple interventions have been implemented such as antibiotic consumption and AMR surveillance, defining guidelines, designing interventional programs to ensure the appropriate prescription of antibiotics (stewardship), informing and communicating to the general population, as well as the developing training programs for healthcare workers [22]. In order to face this issue, in 2017, the first National Action Plan on Antimicrobial Resistance (Piano Nazionale per il Contrasto dell’Antimicrobico-Resistenza, PNCAR 2017–2020) was adopted in Italy [23]. The PNCAR is a multicomponent tool to implement the Italian strategy against AMR. It provides specific objectives and actions, both in human and in veterinary fields, using a one health strategy through the synergy between national, regional, and local levels. In the same year, in the framework of a Regional Action Plan on HAIs and AMR prevention, the Sicilian Health Authority, Sicilian Region (Southern Italy), implemented a surveillance system of antibiotic consumption in the hospital wards as well as at a community level [24]. The monitoring of antibiotic resistance in hospitals has also been developed. The main goals of the regional surveillance system were to test and validate standardized indicators for antibiotic consumption and AMR, to report the trends in the use of such medications over time, and to allow a better assessment of the impact of programs aimed at minimizing antibiotic misuse and the burden of AMR. The aim of the present article is to describe the methodology adopted for the implementation of this regional surveillance system and to report the results of a three-year monitoring period, from 2015 to 2017.

## 2. Materials and Methods 

In 2017, to carry out the recommendation released by the European Centre for Disease Prevention and Control (ECDC) regarding the prevention and control of AMR [25], the PNCAR 2017–2020 [23] working group proposed a coordinated and sustainable approach to tackle the threat of AMR on a national, regional, and local level. The plan indicated the main actions to be implemented at each level to promote effective strategies in the following areas: (i) Surveillance, prevention and control of AMR and infections with resistant microorganisms; (ii) appropriate use and surveillance of antimicrobial consumption; (iii) strengthening of microbiology diagnostic services; (iv) training of healthcare workers; (v) information/education of the general population through communication campaigns; (vi) research and development. 

During 2017, the Sicilian region implemented a regional surveillance system aimed at analyzing the consumption of antibiotics, both in the community and in hospital settings (unit-based surveillance), as well as the development of antibiotic resistant microorganisms (laboratory-based surveillance).

Data for the years 2015, 2016, and 2017, were retrospectively collected in order to test and validate a shared and standardized system of indicators, useful for tracking and monitoring the use of antibiotics and AMR. 

All public and private healthcare facilities (hospitals and other healthcare facilities) were requested to provide antibiotic consumption and antibiotic resistance data for the years 2015, 2016, and 2017 through the online regional surveillance platform created ad-hoc for this purpose [26]. Participation was on a voluntary basis. Data on antibiotic use were obtained retrospectively from the pharmacies of the participating healthcare facilities. Particularly, data—expressed as defined daily dose (DDD), the average maintenance dose per day for a drug used for its main indication in adults —on consumption of tetracyclines, penicillins, other beta-lactam antibacterials, cephalosporins, carbapenems, sulfonamides and trimethoprim, macrolides, lincosamides and streptogramins, quinolones, fluoroquinolones, glycopeptides, and colistin were collected. 

Antibiotic consumption data in the community were retrieved by the Sicilian Health Authority through the Sicilian Regional Healthcare Database which contains all outpatient prescriptions of drugs reimbursable by the National Healthcare System. More specifically, through the use of the marketing authorization code, all medications included in the study were linked to a drug register containing information on the commercial name of the drug, the quantity of the active principle of the drug contained in one box, DDDs of the active principle, and the estimated coverage of one box.

Indicators were calculated for each antibiotic classes by using the WHO Anatomical Therapeutic Chemical (ATC) classification system [27]. Particularly, antibiotic consumption in a hospital was expressed as DDD per 100 patient-days (or per 100 discharges, data not shown); antibiotic consumption in the community was reported as DDD per 1000 inhabitants per day. The 3-year trend of consumption, from 2015 to 2017, was assessed using linear regression.

Resistance data on seven bacterial pathogens commonly causing infections in humans—*E. coli*, *K. pneumoniae*, *P. aeruginosa*, *A. baumannii*, *Streptococcus pneumoniae*, *S. aureus*, and *E. faecium*—were retrospectively collected using routine clinical antimicrobial susceptibility data from microbiological laboratories of participating hospitals. Microbiological identification and routine antibiotic susceptibility testing were performed in each clinical laboratory using different methods depending on the laboratory test that they provided, including the disk diffusion and minimum inhibitory concentration methods and commercial systems, utilizing both phenotypic and genotypic characterization of bacterial resistance. Only microbiological results from blood and cerebrospinal fluid isolates (not repeated within 28 days), which are responsible for invasive infections, were considered. The isolates were classified as resistant to an antibiotic class when they showed non-susceptibility to at least one of the antibiotics belonging to a specific class. For each relevant antibiotic, resistance rates (RRs) were calculated as the number of non-susceptible isolates (i.e. resistant or intermediate isolates) divided by the total number of isolates of the same species tested against the corresponding antibiotic, multiplied by 100. For each indicator related to antibiotic resistance, trends from 2015 to 2017 were evaluated by the Chi Square test.

## 3. Results

### 3.1. Antibiotic Consumption in Healthcare Facilities

**Figure 1:**
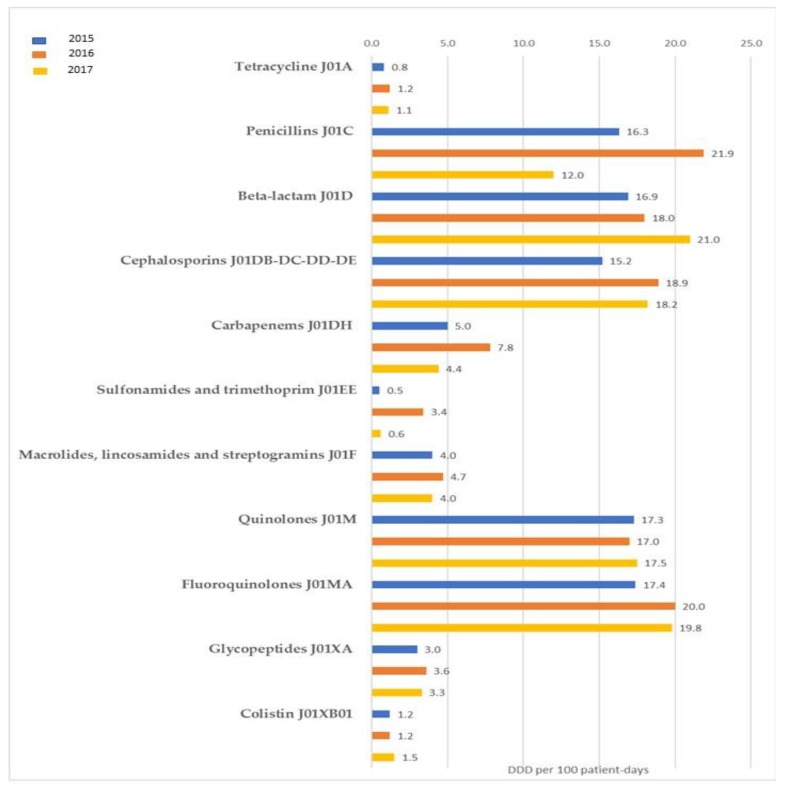
Consumption of antibiotics in healthcare facilities (expressed as Defined Daily Dose per 100 patient-days) by Anatomical Therapeutic Classification groups in 2015 (A), 2016 (B), and 2017 (C), in Sicily (Southern Italy). (All healthcare facilities).

During the three-year period under investigation, data on antibiotic consumption was respectively collected from 91 (in 2015), 92 (in 2016), and 83 (in 2017) private and public healthcare facilities. Antibiotic consumption for the ATC class J01 (i.e., antibacterial for systemic use), was 74.2 DDD per 100 patient days in 2015, 100.7 DDD per 100 patient days in 2016, and 92.3 DDD per 100 patient days in 2017. The most used classes were fluoroquinolones (17.4 DDD per 100 patient days) in 2015, penicillins (21.9 DDD per 100 patient days) in 2016, and other beta-lactam antibacterials (21.0 DDD per 100 patient days) in 2017. Figure 1 shows the yearly consumption of antimicrobials by ATC groups, expressed as DDD per 100 patient days, in all participating healthcare facilities. In comparing consumption data from the 63 healthcare facilities that constantly provided data for the entire surveillance period (2015–2017), there were no significant differences observed, except for the J01D class (other beta-lactam antibacterials) that increased significantly from 17.7 to 19.4 DDD per 100 patient days (*p* = 0.027).

### 3.2. Antibiotic Consumption in the Community

Antibiotic consumption data for J01 class in the community was 23.4 DDD per 1000 inhabitants per day in 2015, 22.5 DDD per 1000 inhabitants per day in 2016, and 22.5 DDD per 1000 inhabitants per day in 2017. Table 1 shows the comparison between the consumption of each J01 class for the 2015–2017 period. During the three-year period, the most consumed class of antibiotics in the community was represented by penicillin. We also evaluated the seasonal variation of antibacterials for systemic use (J01) calculated as the ratio between winter consumption (October–December, January–March) and summer consumption (July–September, April–June) per 100, with a special focus on quinolones. However, no statistically significant variation was observed during the three-year study period in terms of seasonal variation in the consumption of antibacterials as well as for the class of quinolones.

### 3.3. Resistance Rates

Considering only the 63 healthcare facilities that provided data for all three years of surveillance, *K. pneumonia* showed a significant increase in RRs to all antibiotic classes, except to carbapenems. RRs of third-generation cephalosporins and carbapenems *E. coli* showed significant decreasing trends. RRs of the other microorganisms did not change significantly during the study period. Table 2 shows RRs computed using the data provided by public and private health care facilities that provided data for the entire study period.

## 4. Discussion

The onset and the rapid spread of AMR is a global health challenge because it compromises the ability to treat potentially life-threatening infections [28,29]. AMR has reached a very high and dangerous level and this might lead medicine back to a pre-antibiotic era, when it was possible to die for common infections [16,30]. The main drivers of AMR occurrence and spread are the inappropriate use of antimicrobial agents and the transmission of AMR microorganisms. Particularly, antimicrobial use contributes to the emergence and selection of AMR, and poor infection prevention and control strategies further spread these microorganisms [31]. Furthermore, particular attention should be paid in critical settings, such as intensive care units and surgical units, in order to implement effective infection control strategies and improve compliance with antimicrobial prophylaxis practices [9,21,32].

The abuse of antibiotics, not only in humans but also in veterinary and agriculture sectors, the increase of global pollution that contributes to the higher presence of antibiotics in the environment, and the horizontal gene transfer across microorganisms are all considered causes of AMR [33]. The overuse of antibiotics are wide spread, from poor and developing countries to developed countries [34]. As described in the most recent ECDC reports, Italy is one of the European countries that is seeing the increasing spread of AMR microorganisms, especially MDR [31], and with high antibiotic consumption both in the community and in hospital settings [35]. In Italy, a mandatory national surveillance of bloodstream infections due to carbapenemase-producing *K. pneumoniae* and *E.coli* isolates was activated in 2013 [36]. Furthermore, in 2017, the PNCAR was established in order to face the alarming AMR problem using the one-health approach [23].

European Antimicrobial Resistance Surveillance Network (EARS-Net) data for 2017 show wide variations depending on bacterial species, antimicrobial group, and geographical region with generally higher resistance percentages reported from the southern and eastern parts of Europe than from Northern Europe. Several countries reported carbapenem resistance percentages above 10% for *K. pneumoniae* and higher percentages in *P. aeruginosa*, while carbapenem resistance remained a rare event for *E. coli* [31]. In our study, considering only the hospitals that provided data for the entire study period, strains of *K. pneumoniae* resistant to third-generation cephaalosporins, fluoroquinolones, and colistin increased during the three-year period. By contrast, the number of *E. coli* resistant to carbapenems and third-generation cephalosporins decreased. With respect to *P. aeruginosa,* different trends were evident during the three-year period: Strains resistant to piperacillin in combination with tazobactam, ceftazidime, and fluoroquinolones were more prevalent in 2015, while carbapenem-resistant strain decreased in 2016 and increased in 2017. High percentages of AMR *K. pneumoniae* and *A. baumannii* were found, highlighting the continuous loss of an effective antimicrobial therapy against these microorganisms and emphasizing the need for multimodal strategies targeting the prudent use of antibiotics, as previously reported [8].

Data from the European Surveillance of Antimicrobial Consumption Network (ESAC-Net) [35] describe that in the community, the average consumption of antibacterials for systemic use in 2017 was 21.8 DDD per 1000 inhabitants per day in Europe (country range: 10.1–33.6) and Italy has one of the highest rates of antibiotic consumption (23.4 DDD per 1000 inhabitants per day) in Europe. This last value is similar to those reported in our study where no statistically significant change was observed between 2015–2017, while the European report describes statistically decreasing trends for some countries, including Italy. Notably, in the European report [35], the indicator DDD per 1000 inhabitants per day was applied for both the community and the hospital sector, which did not allow for a comparison with our data for the hospital sector. In all European countries, the consumption of major subgroups of antibiotics shows that, in the community in 2017, penicillin was the most used antibacterial. In Italy, the consumption of penicillin was 13.1 DDD per 1000 inhabitants per day in 2017, while in our study, in Sicily the consumption was 11.5 DDD per 1000 inhabitants per day. In Italy, as well as in Spain, Luxemburg, Bulgaria, and other countries, with a higher use in Cyprus, the consumption of quinolones was higher than in other countries with a consumption of 2.7 DDD per 1000 inhabitants per day, showing a gap with our data from Sicily, which had a consumption of 3.5 DDD per 1000 inhabitants per day. Macrolides, lincosamides, and streptogramins were the second most used classes of antibiotics in Europe in 2017. In Sicily, as well as for quinolones, the consumption was higher than Italy and Europe, with a DDD per 1000 inhabitants per day of 4.3, while in Italy 3.8 and in Europe 2.9. Considering data of consumption of other beta-lactams, in Italy and in Sicily, the DDD per 1000 inhabitants per day was, respectively, 1.9 and 2.3, showing that the use of beta-lactams is in accordance with Europe, where the consumption was 2.0 DDD per 1000 inhabitants per day. Although Italy is one of the countries with a higher antibiotics consumption, Spain, Cyprus, France, Romania, and other countries had very relevant data for the total consumption in 2017 [31]. 

A recent study by Mor et al., conducted in a large population of 29 million citizens from five European countries showed a higher prevalence of the use of antibiotic drugs in Italy as compared to other countries, with a particularly large consumption of broad-spectrum medications [37]. 

The seasonal variability (although not significant) in the use of antimicrobial medications observed in our study is also in line with another investigation that focused on macrolide use in the pediatric population, where a marked seasonality in the prescription rates of such medications has been observed in Italy, thus suggesting a frequent prescription of antibiotics to treat respiratory infections which might have a viral origin [38].

## 5. Conclusions

The Global Action Plan on AMR of the WHO recognizes the surveillance of antibiotic consumption and resistance in humans and animals as a key strategy to address AMR. In general, the surveillance of antibiotic consumption can allow one to identify targets for interventions, as well as changing trends in antimicrobial usage patterns [29,39]. Accordingly, the regional surveillance system implemented in Sicily, which describes trends of antibiotic consumption both in the hospital sector and in the community and antibiotic resistance, help to identify targets for effective strategies against AMR. In order to face the problem, the Sicilian region founded and implemented a multifaceted program, the Regional Programme for the reduction of Catheter related Bloodstream Infections, “Targeting Zero” [40], which integrates surveillance and antimicrobial stewardship policies with infection prevention and control activities, through the promotion of clinical guidelines targeting the reduction of catheter-associated bloodstream infections, participation to repeated point prevalence surveys on HAIs, and antimicrobial use in hospitals. Furthermore, an educational campaign called “Obiettivo Antibiotico—Antibiotic Aim” (https://www.obiettivoantibiotico.it/) was designed and launched to raise awareness on the prudent use of antibiotics in the public and in healthcare professionals in Sicily, using toolkits of the European Antibiotic Awareness Day (EAAD) initiative of the ECDC adapted using regional surveillance data of AMR and antibiotic consumption. The implementation of the Sicilian surveillance system provides Sicilian reference data, both in the community and the hospital sector, to monitor trends and target interventions and policies for reducing the threat of AMR. 

## Figures and Tables

**Table 1:** Antibiotic consumption in the community during the three-year period (2015–2017).

Consumption of Antibiotics for Systemic Use (J01) per 1000 Inhabitants Per Day	2015	2016	2017	*p*-Trend
Antibacterials for systemic use (J01)	23.4	22.5	22.5	0.667
Penicillin (J01C)	11.6	11.3	11.5	0.879
Other beta-lactams (J01D)	2.6	2.5	2.3	0.212
Cephalosporins (J01DB-DC-DD-DE)	2.6	2.5	2.3	0.212
Macrolides, lincosamides, and streptogramins (J01F)	4.6	4.3	4.3	0.407
Quinolones (J01M)	3.8	3.5	3.5	0.667
Fluoroquinolones (J01MA)	3.8	3.5	3.5	0.667

**Table 2:** Resistance rates of included microorganisms during the three-year period (2015–2017).

Microorganism	Antibiotic	Resistance Rates			*p*-Trend *
		2015	2016	2017	
*K. pneumoniae*	Third-generation cephalosporins	**70.3**	**73.0**	**81.3**	**<0.001**
	Carbapenems	44.3	48.9	45.0	0.838
	Fluoroquinolones	**71.5**	**73.0**	**83.8**	**<0.001**
	Colistin	**13.6**	**15.2**	**20.9**	**<0.001**
*E. coli*	Third-generation cephalosporins	**46.5**	**42.4**	**38.7**	**<0.001**
	Carbapenems	**4.8**	**1.9**	**1.6**	**<0.001**
	Fluoroquinolones	58.9	56.4	61.0	0.510
*P. aeruginosa*	Carbapenems	**33.1**	**25.9**	**43.3**	**0.031**
	Ceftazidime, Fluoroquinolones, Piperacillin + Tazobactam	**39.3**	**35.7**	**27.5**	**0.004**
*A. baumannii*	Carbapenems	86.8	85.9	86.9	0.982
*S. aureus*	Methicillin	55.8	55.0	54.2	0.626
*S. pneumoniae*	Erythromycin	38.5	35.3	50.0	0.461
	Penicillin	27.3	28.6	33.3	0.715
*E. faecium*	Vancomycin	8.3	9.1	7.5	0.849

* Statistically significant trends are indicated in bold font.
